# Protein Kinase Ime2 Is Required for Mycelial Growth, Conidiation, Osmoregulation, and Pathogenicity in Nematode-Trapping Fungus *Arthrobotrys oligospora*

**DOI:** 10.3389/fmicb.2019.03065

**Published:** 2020-01-14

**Authors:** Meihua Xie, Na Bai, Jiangliu Yang, Kexin Jiang, Duanxu Zhou, Yining Zhao, Dongni Li, Xuemei Niu, Ke-Qin Zhang, Jinkui Yang

**Affiliations:** ^1^State Key Laboratory for Conservation and Utilization of Bio-Resources in Yunnan, Yunnan University, Kunming, China; ^2^School of Life Sciences, Yunnan University, Kunming, China; ^3^Key Laboratory for Microbial Resources of the Ministry of Education, Yunnan University, Kunming, China; ^4^Department of Chemistry and Life Science, Chuxiong Normal University, Chuxiong, China

**Keywords:** *Arthrobotrys oligospora*, inducer of meiosis 2, mycelial development, conidiation, osmolarity, pathogenicity

## Abstract

Inducer of meiosis 2 (Ime2), a protein kinase that has been identified in diverse fungal species, functions in the regulation of various cellular processes, such as ascospore formation, pseudohyphal growth, and sexual reproduction. In this study, AoIme2, an ortholog of *Saccharomyces cerevisiae* Ime2, was characterized in the nematode-trapping fungus *Arthrobotrys oligospora*. Disruption of the gene *Aoime2* caused defective growth, with slower mycelial growth in Δ*Aoime2* mutants than the wild type (WT) strain, and in the mutants, the number of hyphal septa in mycelia was higher and the number of cell nuclei in mycelia and conidia was considerably lower than in the WT strain. The conidial yields of the Δ*Aoime2* mutants were decreased by ∼33% relative to the WT strain, and the transcription of several sporulation-related genes, including *abaA*, *fluG*, *rodA*, *aspB*, *velB*, and *vosA*, was markedly downregulated during the conidiation stage. The Δ*Aoime2* mutants were highly sensitive to the osmotic stressors NaCl and sorbitol, and the cell wall of partial hyphae in the mutants was deformed. Further examination revealed that the cell wall of the traps produced by Δ*Aoime2* mutants became loose, and that the electron-dense bodies in trap cells were also few than in the WT strain. Moreover, *Aoime2* disruption caused a reduction in trap formation and serine-protease production, and most hyphal traps produced by Δ*Aoime2* mutants did not form an intact hyphal loop; consequently, substantially fewer nematodes were captured by the mutants than by the WT strain. In summary, an Ime2-MAPK is identified here for the first time from a nematode-trapping fungus, and the kinase is shown to be involved in the regulation of mycelial growth and development, conidiation, osmolarity, and pathogenicity in *A. oligospora*.

## Introduction

Mitogen-activated protein kinase (MAPK) cascades function as key intracellular signal transducers that use protein phosphorylation/dephosphorylation cycles to transmit information, and orthologous MAPK signaling modules in yeast and filamentous fungi have been found to be involved in regulating mating, filament growth, hyperosmotic-stress response, cell-wall integrity (CWI), and spore-wall assembly (Xu, 2000; Zhao et al., 2007; Rispail et al., 2009). MAPK is generally activated by phosphorylation at the well-conserved threonine-x-tyrosine (TXY) motif by MAPK kinase (MAPKK), which is in turn activated by MAPKK kinase (MAPKKK) (Yang et al., 2003; Jiang et al., 2018). These MAPKKK–MAPKK–MAPK cascades are evolutionarily conserved in eukaryotes (Herskowitz, 1995; Schaeffer and Weber, 1999). In *Saccharomyces cerevisiae*, five MAPK pathways (Kss1, Fus3, Hog1, Slt2, and Smk1 pathways) have been identified and shown to regulate mating, invasive growth, CWI, osmolarity, and ascospore formation (Jiang et al., 2018), and in filamentous fungi, three MAPK pathways corresponding to pathways in *S. cerevisiae* have been reported: the Fus3/Kss1-homolog, Slt2-homolog, and Hog1-homolog pathways (Jiang et al., 2018). Moreover, a fourth MAPK pathway, the inducer of meiosis 2 (Ime2)-homolog pathway, was found in several fungi, such as *Aspergillus nidulans* (Bayram et al., 2009), *Cryptococcus neoformans* (Liu and Shen, 2011), *Neurospora crassa* (Hutchison and Glass, 2010), and *Ustilago maydis* (Garrido et al., 2004). Unlike in the three classic MAPK pathways, the MAPKKK and MAPKK in the Ime2-MAPK cascade remain unidentified, and thus these kinases belong to a distinct Ime2-MAPK class (Garrido and Pérez-Martín, 2003; Schindler et al., 2003). Ime2 homologs are conserved in not only in fungi, but also all eukaryotic taxa examined (Krylov et al., 2003), and the common feature of these kinases is that their *N*-terminal region harbors a TXY motif, which is typically found in the activation loop of MAPKs (Payne et al., 1991).

The *ime2* was first identified in *S. cerevisiae* as a gene that is expressed exclusively during meiosis (Smith and Mitchell, 1989; Yoshida et al., 1990), and, subsequently, was shown to be also involved in normal spore formation (Sari et al., 2008) and pseudohyphal growth (Strudwick et al., 2010). Recently, Ime2 homologs from various fungal species have been increasingly shown to function in not only the control of meiosis, but also the regulation of diverse cellular processes, including ascospore formation, pseudohyphal growth, and sexual reproduction in response to light and nutrient deprivation (Irniger, 2011). For example, Crk1, a homolog of yeast Ime2, was first identified in *U. maydis* to participate in the regulation of morphogenesis and plant infection (Garrido et al., 2004), and later reported to function in the negative regulation of mating in *C. neoformans* (Liu and Shen, 2011). The Ime2 homolog ImeB was found to be not required for meiosis in *A. nidulans*, but vegetative growth of the Δ*imeB* mutants was diminished, and fully fertile ascospores were produced in morphologically normal cleistothecia (Bayram et al., 2009). Moreover, an Ime2-like MAPK was shown to be involved in cellulase expression in *Trichoderma reesei* (Chen et al., 2015). Therefore, the conserved Ime2-family proteins display an unexpected diversification in their cellular functions in fungi.

Nematode-trapping (NT) fungi constitute a group of fungi that can capture nematodes; NT fungi develop specific trapping devices (traps) for nematode predation (such as adhesive networks, adhesive knobs, and constricting rings), are widely distributed in terrestrial and aquatic ecosystems, and survive mainly as saprophytes and enter a predacious phase in response to signals released by nematodes (Ahrén et al., 1998; Nordbring-Hertz et al., 2001; Su et al., 2017). The traps produced by NT fungi harbor numerous electron-dense (ED) bodies, but normal vegetative hyphae lack ED bodies (Veenhuis et al., 1985, 1989). Traps are critical tools used by NT fungi and their integrity affects the nematode-predation efficiency of the fungi. Moreover, NT fungi produce extracellular serine proteases that can degrade the nematode cuticle and thereby facilitate fungal penetration and colonization (Tunlid et al., 1994; Yang et al., 2013). Therefore, NT fungi are potential agents for controlling parasitic nematodes of plants and animals.

*Arthrobotrys oligospora*, a common NT-fungus species that has been isolated from diverse soil and aquatic environments, can produce adhesive traps (three-dimensional networks) to capture nematodes (Nordbring-Hertz, 2004; Yang et al., 2011). We have sequenced the genome of *A. oligospora* (Yang et al., 2011) and identified several signaling proteins involved in *A. oligospora* growth, conidiation, and pathogenicity, such as the MAPK protein AoSlt2 (Zhen et al., 2018) and the Rab protein AoRab-7A (Yang et al., 2018). However, little is known regarding the role of the Ime2-homolog MAPK in NT fungi. Here, we identified AoIme2, an ortholog of *S. cerevisiae* Ime2 in *A. oligospora*, and characterized by constructing deletion mutants of *Aoime2*. Our results suggest that AoIme2 plays crucial roles in regulating hyphal growth, cell nucleus development, conidiation, trap formation, and pathogenicity in *A. oligospora*.

## Materials and Methods

### Fungal Strains and Culture Conditions

*Arthrobotrys oligospora* Fres. (ATCC 24927) and Δ*Aoime2* mutants were cultured on potato dextrose agar (PDA) plates at 28°C. *S. cerevisiae* strain FY834 used for constructing recombinant plasmid vectors was cultured in yeast extract peptone dextrose (YPD) medium. Plasmids pRS426 and pCSN44 were maintained in *Escherichia coli* strain DH5α (TaKaRa, Shiga, Japan). Protoplasts of *A. oligospora* were regenerated on PDASS medium (PDA supplemented with 10 g/L molasses and 0.4 M saccharose) containing hygromycin (200 μg/mL). CMY (20 g/L maizena, 5 g/L yeast extract, 20 g/L agar), TG (10 g/L tryptone, 10 g/L glucose, 20 g/L agar), and TYGA (10 g/L tryptone, 5 g/L yeast extract, 10 g/L glucose, 5 g/L molasses, 20 g/L agar) media were prepared as previously described (Yang et al., 2018) and used for analyzing mycelial growth and related phenotypic traits. *Caenorhabditis elegans* (strain N2) worms were cultured on oatmeal medium at 26°C and then used for bioassays.

### Analyses of AoIme2 Sequence and Phylogenetic Tree

AoIme2 sequence (AOL_s00188g140) was retrieved by performing BLASTP searches^1^ with queries for orthologous Ime2 proteins of the model fungi *S. cerevisiae*, *A. nidulans*, and *N. crassa* in the NCBI database. The theoretical isoelectric point and molecular weight of AoIme2 were calculated using a pI/MW tool^2^. Ime2 orthologs in fungi were searched for by using BLAST algorithm, and the similarity of the orthologs from different fungi was examined by aligning them by using DNAman software package (version 5.2.2; Lynnon Biosoft, San Ramon, CA, United States) and then performing phylogenetic analysis by using MEGA 7.0 software (Kumar et al., 2016).

### Generation of *Aoime2* Mutants

*Aoime2* was disrupted through homologous recombination as previously described (Tunlid et al., 1999; Colot et al., 2006). All primers used in this study are described in Supplementary Table S1. The upstream and downstream fragments of the *Aoime2* were, respectively, PCR-amplified from *A. oligospora* by using paired primers (Supplementary Table S1), and the hygromycin-resistance gene cassette (*hph*) was amplified using pSCN44 plasmid as the template. Next, the three DNA fragments and a linearized pRS426 vector were cotransformed into *S. cerevisiae* strain FY834 cells through electroporation, and after isolating the recombinant plasmid pRS426-AoIme2-hph from the yeast, the complete target sequence was amplified using primers AoIme2-5f and AoIme2-3r (Supplementary Table S1) and transformed into protoplasts of *A. oligospora* as described (Zhen et al., 2019). Colonies grown on PDASS medium containing 200 μg/mL hygromycin B were further verified using PCR and Southern blotting analyses. Southern blotting was performed using a North2South chemiluminescent hybridization and detection kit (Pierce, Rockford, IL, United States), according to the manufacturer’s instructions. Genomic DNA was extracted from WT and Δ*Aoime2* mutant strains by using a plant genomic DNA Kit (TaKaRa) and digested with *Kpn*I for Southern blotting analysis.

### Analyses of Mycelial Growth, Conidiation, and Morphology

Wild type (WT) and mutant strains were cultured on PDA plates at 28°C for 6 days, and then a 7-mm-diameter hyphal disk from each strain was, respectively, inoculated on PDA, TYGA, and TG plates at 28°C for 3–7 days; mycelial growth rate and colony morphology were examined and quantified at specific time intervals (Xie et al., 2019). WT and mutant strains were also incubated on CMY medium at 26°C for 15 days, and the conidial yield was determined as previously described (Liu et al., 2017). For observation of the septum in mycelia and conidia, freshly harvested conidia and hyphae of WT and mutant strains were stained with 20 μg/mL calcofluor white (CFW) (Sigma-Aldrich, St. Louis, MO, United States), and mycelial and conidial cell nuclei were visualized by staining with 20 μg/mL DAPI and 20 μg/mL CFW as described (Li et al., 2015); samples were analyzed using an inverted fluorescence microscope (Carl Zeiss, Heidenheim, Germany). Hyphal and conidial morphology was examined using scanning electron microscopy (SEM), and the ED bodies of trap cells in WT and mutant strains were examined using transmission electron microscopy (TEM). Samples were processed for SEM and TEM as previously described (Zhang et al., 2013, 2019).

### Analysis of Tolerance to Chemical Stresses

Resistance to chemical stresses was evaluated by measuring the relative growth inhibition (RGI) values of fungal colonies on TG medium alone (control) or medium supplemented with reagents that induce chemical stresses, such as H_2_O_2_ and menadione for oxidative stress, NaCl and sorbitol for osmotic pressure, and SDS and Congo red for cell-wall-perturbing stress (Liu et al., 2017). The colonies were examined, and the diameter of each colony was measured. These experiments were performed in triplicate.

### Trap Formation and Bioassay

Freshly harvested conidia (2 × 10^4^) of WT and mutant strains were evenly spread on WA (water agar, 20%) medium and incubated at 28°C for 3–4 days, and ∼300 nematodes were added to each WA plate to induce trap formation. Traps and captured nematodes were examined and quantified (per plate) under a microscope (Olympus, Tokyo, Japan) at 12-h intervals. The experiments were performed in triplicate.

### Proteolytic-Activity Assays

Wild type and mutant strains were cultured in PL-4 liquid medium (Yang et al., 2011) at 28°C for 6 days, after which the fermentation broth was collected and proteolytic activity was qualitatively assayed on milk-plate medium (2% skimmed milk powder) as previously described (Zhao et al., 2004). Protease activity was also quantified as described (Wang et al., 2006). The effect of a protease inhibitor on enzyme activity and hyphal biomass was determined as described (Xie et al., 2019). Moreover, WT and mutant strains were incubated in PL-4 liquid medium at 28°C, followed by 3, 5, or 7 days incubation to collect hyphae for RNA extraction. Seven genes encoding serine proteases belonged to different subfamilies in *A. oligospora* were selected, and their transcriptional levels were analyzed in WT and mutant strains as described (Yang et al., 2018).

### Quantitative Real-Time PCR (RT-PCR) Analysis

To analyze the expression of sporulation-related genes in WT and mutant strains, total RNAs were extracted from 3-, 5-, and 7-day cultures grown on TYGA by using an AxyPrep multisource RNA miniprep kit (Axygen, Jiangsu, China), and then reverse-transcribed into cDNAs by using a FastQuant RT kit with gDNase (TaKaRa). The cDNA samples of each strain were used as the template to assess the transcript level of each gene by using SYBRH Premix Ex Taq (TaKaRa) and performing RT-PCR with paired primers (Supplementary Table S2); β-tubulin served as an internal standard, and the 2^–ΔΔCt^ method (Livak and Schmittgen, 2001) was used for quantification. The relative transcription level (RTL) of each gene was calculated as the ratio of the transcription level in the deletion mutant to the transcription level in the WT strain at a given time point.

### Statistical Analysis

Data are presented as means ±standard deviation (SD). SPSS program (version 16.0) (SPSS, Inc., Chicago, IL, United States) was used to analyze data from triplicate experiments, and *p* < 0.05 was used as the threshold for determining significant differences.

## Results

### Sequence and Phylogenetic Analyses of AoIme2

The gene *Aoime2* encodes a 790-aa polypeptide featuring an isoelectric point of 10.29 and a molecular mass of 86.91 kDa. AoIme2 contains a conserved protein kinase domain (IPR000719), an active site (IPR008271), and an ATP-binding site (IPR017441). For phylogenetic analysis, we used 26 proteins orthologous to Ime2 and classic MAPK-family proteins (Kss1/Fus3, Slt2, and Hog1) from diverse fungi; the constructed neighbor-joining phylogenetic tree revealed that these MAPK-family proteins were divided into two clades (A and B) (Supplementary Figure S1). Ime2 orthologs from different fungi are clustered in Clade A, with the Ime2 orthologs from NT fungi being clustered in Subclade A-II; conversely, the classic MAPK-family members from different fungi are clustered in Clade B, which is further divided into three subclades: Fus3, Slt2, and Hog1 (Supplementary Figure S1). The similarity of orthologous Ime2 and MAPKs from different fungi was analyzed using DNAman software, and AoIme2 sequence was found to share 88.8 and 86.4% identity, respectively, with the sequences from the NT fungi *Dactylellina haptotyla* (Meerupati et al., 2013) and *Drechslerella stenobrocha* (Liu et al., 2014). The sequence identity between AoIme2 and the orthologs from other filamentous fungi reaches 49.9–51.7%, and the lowest identity is with the *S. cerevisiae* ortholog, 22.9%. Moreover, AoIme2 shares only 18.8–26.6% sequence similarity with fungal classic MAPKs. The Ime2 orthologs from various fungi contain two conserved motifs: the protein kinase active-site motif “-D[L/I/V]K-,” and the phosphorylation site “-TXY-” located in the activation loop. The phosphorylation-site sequences in orthologous Fus3 and Slt2 are both “-TEY-”, the sequence in Hog1 is “-TGY-”, and that in Ime2 from different fungi is “-TTY-” except in the case of ScIme2 (from *S. cerevisiae*) (Supplementary Figure S2).

### Verification of Positive Transformants

*Aoime2* was disrupted by inserting the *hph* cassette, and transformants were selected on PDASS medium containing hygromycin B (Tunlid et al., 1999). Genomic DNA was isolated from the WT strain and transformants and used as the template for PCR amplification with primers yz140-5f and yz140-3r (Supplementary Table S1); the sizes of the fragments amplified from the WT strain and transformants were consistent with the expected sizes, respectively, 3,044 and 2,207 bp (Supplementary Figures S3A,B). Subsequently, the genomic DNA of the WT strain and transformants was digested with the restriction enzyme *Kpn*I and used in Southern blotting analysis (Supplementary Figure S3A), which revealed a single band, of the expected size, representing the DNA that hybridized with the *Aoime2* probe in the case of both the WT strain and the transformants (Supplementary Figure S3C). Ultimately, two positive transformants (MT-1 and MT-14) were obtained based on the PCR and Southern blotting analyses.

### AoIme2 Regulates Mycelial Growth, Morphology and Cell Nucleus Development

**FIGURE 1:**
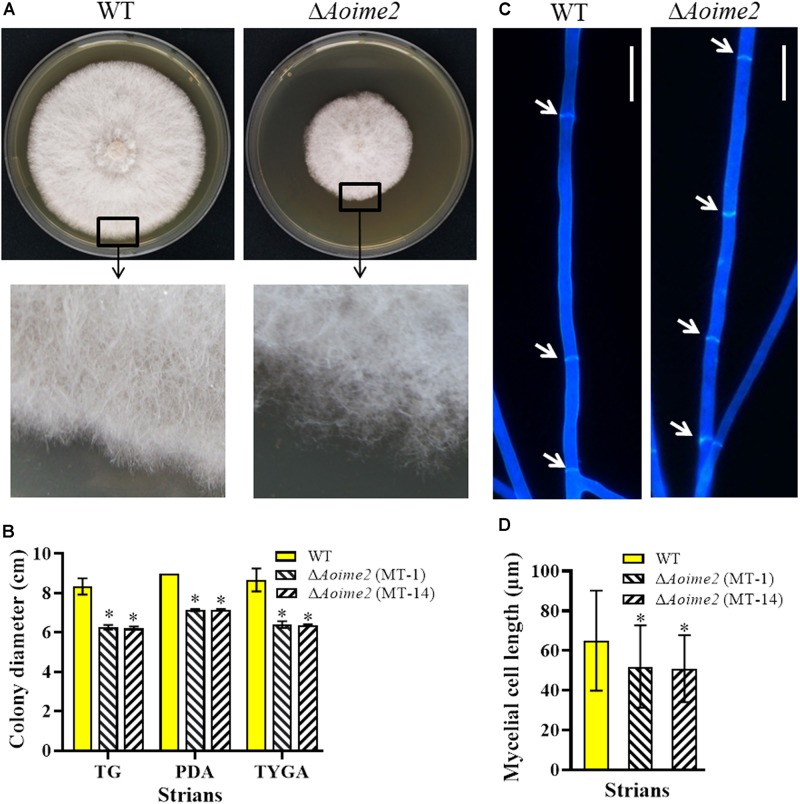
Comparison of mycelial growth, morphology, and septum formation between wild type (WT) and Δ*Aoime2* mutants. **(A)** Colony morphology (upper panel) and hyphae (lower panel) of WT and Δ*Aoime2* mutant strains incubated on TYGA medium for 5 days at 28°C. **(B)** Colony diameters of WT and Δ*Aoime2* mutants incubated on PDA, TYGA, and TG media for 7 days. **(C)** Hyphal septa of WT and mutants were stained with 20 μg/mL calcofluor white (CFW) after the fungal strains were incubated on CMY medium for 7 days. Arrows: hyphal septa. Bar = 10 μm. **(D)** Comparison of mycelial cell size of WT and mutants; 60 mycelial cells were randomly selected, and the distance between two hyphal septa was measured using ImageJ software. Error bars: SD from 60 replicates; asterisk: significant difference between mutant and WT (Tukey’s HSD, *p* < 0.05).

The WT strain and the two Δ*Aoime2* mutants (MT-1 and MT-14) were cultured on PDA, TG, and TYGA media at 28°C. Mycelial growth rates of each fungal strain did not differ markedly on these media, but the mycelial growth rate of the Δ*Aoime2* mutants was significantly lower than that of the WT strain on all three media (Figures 1A,B). The WT strain produced extremely dense mycelia when grown on TYGA medium, and its aerial mycelia grew robustly. By contrast, the Δ*Aoime2* mutant colonies became loose and the aerial hyphae became sparse (Figures 1A,B). Moreover, the hyphae of the Δ*Aoime2* mutants contained 20% more hyphal septa than WT hyphae (Figure 1C), and thus the hyphal cells of the mutants were shorter than those of the WT strain (Figure 1D).

**FIGURE 2:**
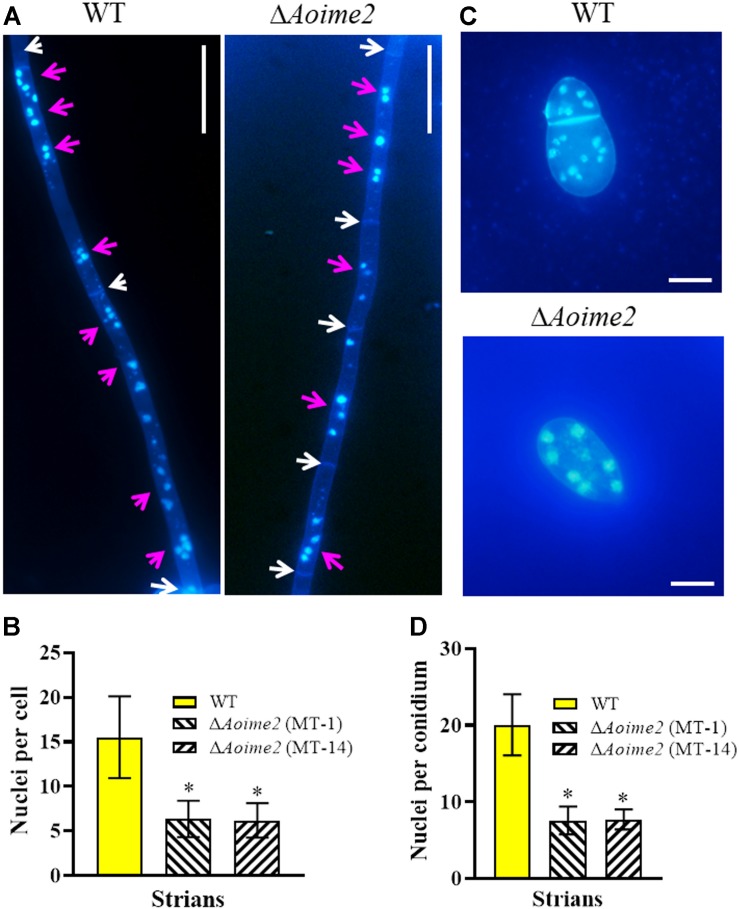
Mycelial and conidial cell nuclei in WT versus Δ*Aoime2* mutant strains. **(A)** Hyphae of WT and Δ*Aoime2* mutant strains were stained with CFW and DAPI after the fungal strains were grown for 7 days on CMY medium; samples were examined using an inverted fluorescence microscope. White arrows: septa; pink arrows: cell nuclei, the strong stained and bright points are identified as nuclei. Bar = 20 μm. **(B)** Comparison of mycelial cell nuclei between WT and mutant strains; 30 mycelial cells were randomly selected and the nucleus in each cell was counted separately. **(C)** Conidia of WT and Δ*Aoime2* mutant strains were stained with CFW and DAPI. Bar = 10 μm. **(D)** Comparison of conidial cell nuclei between WT and mutant strains; 30 conidia were randomly selected for counting cell nuclei. Error bars: SD from 30 replicates; asterisk: significant difference between mutant and WT (Tukey’s HSD, *p* < 0.05).

Nematode-trapping fungi are unique hyphomycetes containing multiple cell nuclei (Nordbring-Hertz, 2004). To determine whether AoIme2 regulates cell nucleus development, we used DAPI to stain cell nuclei in WT and Δ*Aoime2* mutant strains. Whereas the hyphal cells of the WT strain contained 6–22 nuclei, these cells in Δ*Aoime2* mutants contained 4–12 nuclei (Figures 2A,B). Moreover, the conidial cells of the WT strain contained 13–25 nuclei, but only 4–10 cell nuclei were present in the conidia of Δ*Aoime2* mutants (Figures 2C,D).

### AoIme2 Regulates Conidiation and Conidial Morphology

**FIGURE 3:**
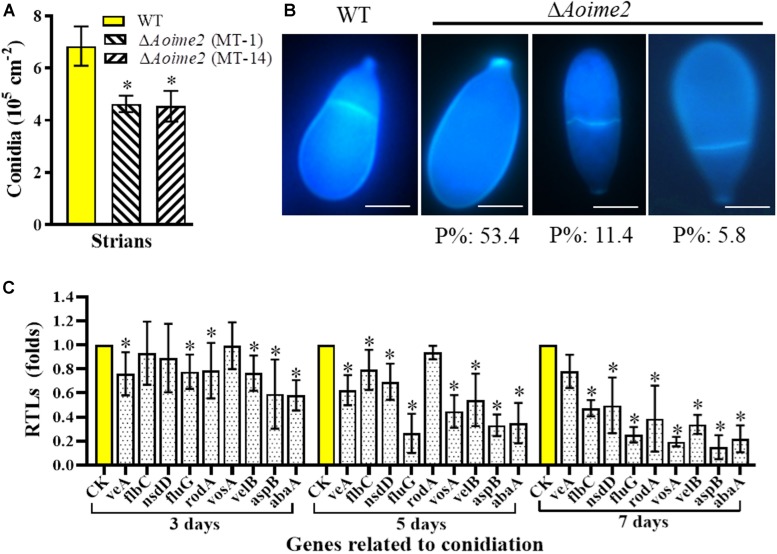
Comparison of conidial yields, morphology, and transcriptional levels (RTLs) of sporulation-related genes between WT and mutant strains. **(A)** Comparison of conidial yields between WT and Δ*Aoime2* mutants. Conidial yields were determined after incubation on CMY medium for 15 days. **(B)** Conidia were stained with CFW and examined under a microscope. Bar = 10 μm. P%: the percentage of each abnormal spore in the Δ*Aoime2* mutant. **(C)** Comparison of RTLs of sporulation-related genes between WT and Δ*Aoime2* mutants at different time points. *CK*, featuring an RTL of 1, was used as the standard for statistical analysis of the RTL of each gene in the deletion mutants to that in the WT strain under a given condition. Error bars: SD from three replicates, asterisk: significant difference between mutant and WT (Tukey’s HSD, *p* < 0.05).

*Aoime2* deletion markedly affected conidiation, with the Δ*Aoime2* mutants producing fewer conidiophores than the WT strain on CMY medium. Furthermore, the partial conidiophores of the *Aoime2* mutant strains produced branches, but similar branches were not detected in WT conidiophores (Supplementary Figure S4). Analysis of 15-day-old cultures on CMY medium revealed that the conidial yields from the WT and Δ*Aoime2* mutant strains were, respectively, 6.6–7.7 × 10^5^ and 4.3–5.2 × 10^5^ conidia cm^–2^ (Figure 3A), the yields of the mutants being decreased by 33.0% relative to WT. *Aoime2* deletion also noticeably altered conidial morphology. For example, the WT strain produced an obovoid spore, with one septum formed near the base of the spore; by contrast, most of the conidia (70.6%) of the Δ*Aoime2* mutants were morphologically abnormal, with 53.4% of the conidia lacking a septum, 11.4% of the conidial septa formed in the middle of the spore, and 5.8% of the conidia elongated in shape (Figure 3B).

From the *A. oligospora* genome, 9 sporulation-related genes were retrieved – *abaA*, *flbC*, *fluG*, *nsdD*, *rodA*, *aspB*, *veA*, *velB*, and *vosA* – based on their homologous genes from the model fungus *A. nidulans* (Krijgsheld et al., 2013), and the transcription of the genes was analyzed using RT-PCR. Most of these genes were expressed at lower levels in Δ*Aoime2* mutants than in the WT strain, and the transcription of the genes in the Δ*Aoime2* mutants was downregulated during conidiation relative to that in vegetative-growth stages. The expression levels of 8 genes, *abaA*, *flbC*, *fluG*, *nsdD*, *rodA*, *aspB*, *velB*, and *vosA*, were substantially downregulated during the conidiation stage in the Δ*Aoime2* mutants (Figure 3C).

### AoIme2 Involves in the Regulation of Osmoregulation

**FIGURE 4:**
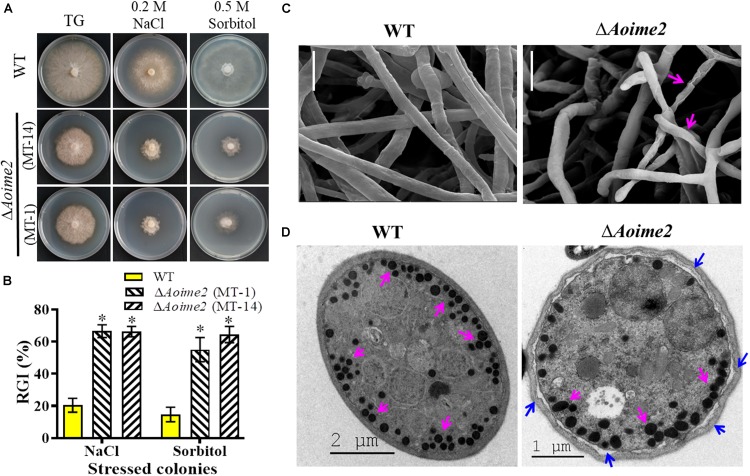
Tolerance to hyperosmotic stress and mycelial morphology: WT versus Δ*Aoime2* mutants. **(A)** Comparison of colonial morphology under high-osmolarity stress between WT and Δ*Aoime2* mutants. **(B)** RGI values of WT and Δ*Aoime2* mutants grown on TG plates supplemented with 0.2 M NaCl or 0.5 M sorbitol. Error bars: SD from three replicates, asterisk: significant difference between mutant and WT strain (Tukey’s HSD, *p* < 0.05). **(C)** Mycelia of WT and Δ*Aoime2* mutant strains were examined using scanning electron microscopy (SEM). Pink arrows: deformed mycelia in Δ*Aoime2* mutants. Bar = 10 μm. **(D)** Traps produced by WT and Δ*Aoime2* mutant strains were examined using transmission electron microscopy. Pink arrows: ED bodies in trap cells of WT and Δ*Aoime2* mutant strains; blue arrows: separation of cell wall and plasma membrane in Δ*Aoime2* mutants.

To investigate whether AoIme2 regulates the biological responses to stresses in *A. oligospora*, we incubated the WT and Δ*Aoime2* mutant strains on media containing different concentrations of chemical agents and then analyzed their responses to these stressors. Mycelial growth of Δ*Aoime2* mutants relative to WT was strongly inhibited on TG medium supplemented with 0.5 M sorbitol or 0.2 M NaCl (Figure 4A); exposure to sorbitol and NaCl increased the RGI values of the Δ*Aoime2* mutants by 40.4–49.7% and 45.8–46.1%, respectively, as compared with the WT strain (Figure 4B). However, no significant difference was measured in the RGI values between WT and Δ*Aoime2* mutant strains on TG media supplemented with oxidants or cell-wall-perturbing agents (Supplementary Figure S5). Furthermore, the mycelial surface of the Δ*Aoime2* mutants was smoother than that in the WT strain and partial hyphae were deformed and shriveled (Figure 4C), and the cell wall of the trap cells of the Δ*Aoime2* mutants became loose, and considerably fewer trap-cell ED bodies were present in the mutants than in the WT strain (Figure 4D).

### AoIme2 Plays a Crucial Role in Trap Formation and Pathogenicity

**FIGURE 5:**
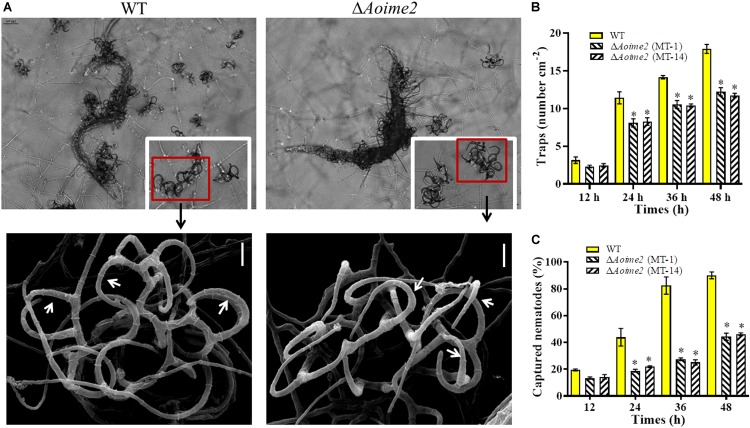
Trap formation, trap morphology, and nematicidal activity of WT and Δ*Aoime2* mutants. **(A)** Trap formation and morphology (upper images: light microscopy; lower images: SEM) of WT and mutants after 48-h induction of traps by nematodes. Red boxes: traps produced by WT and Δ*Aoime2* mutants; arrows: hyphal loops in traps. **(B)** Comparison of traps produced by WT and mutants at 12, 24, 36 and 48 h. **(C)** Percentages of nematodes captured by WT and mutant strains at different time points. Bar = 10 μm. Error bars: SD from three replicates, asterisk: significant difference between mutant and WT (Tukey’s HSD, *p* < 0.05).

The WT and mutant strains were incubated on WA medium at 28°C for 3 days, and trap formation was examined at 12 h after adding nematodes. The WT strain began to produce fresh traps containing 1–2 hyphal loops at 12 h, and mature traps consisting of multiple hyphal loops appeared at 24 h after addition of the nematodes (Figure 5A). Most of the nematodes were captured by the WT strain at 36 h and were digested at 48 h. By comparison, diminished trap formation was observed in the Δ*Aoime2* mutants at all corresponding time points, and most of the hyphal loops were not closed (Figure 5A). At 24, 36, and 48 h after nematode addition, the WT strain produced 11.4, 14.1, and 17.9 traps cm^–2^, respectively, whereas the Δ*Aoime2* mutants produced only 8.1, 10.4, and 12.2 traps cm^–2^ (Figure 5B). Thus, 43.8, 82.5, and 90.1% of the nematodes were captured by the WT strain at 24, 36, and 48 h, respectively, but only 18.8–21.9%, 25.1–27.5%, and 44.3–46.1% were captured by the Δ*Aoime2* mutants at the corresponding time points (Figure 5C).

### AoIme2 Involves in the Regulation of Serine-Protease Production

**FIGURE 6:**
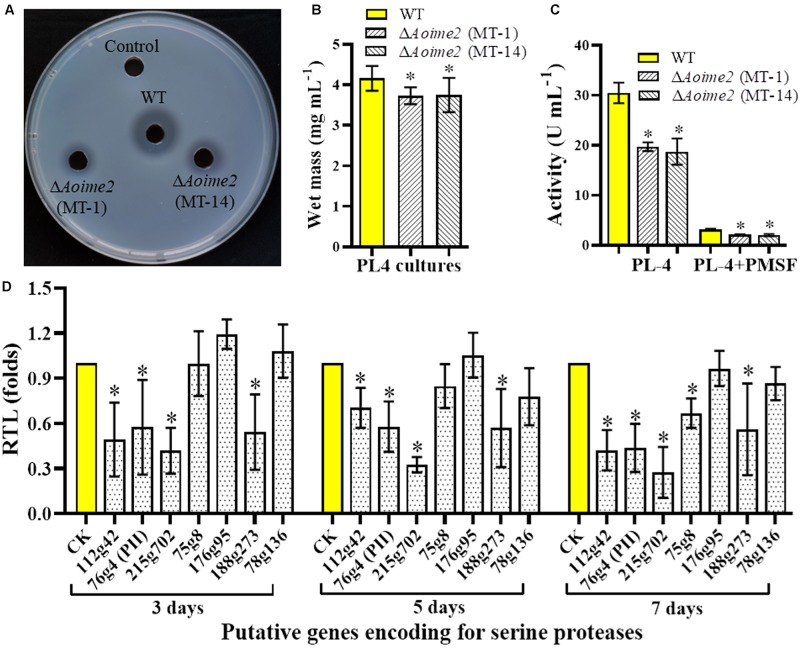
Comparison of extracellular proteolytic activity and transcriptional levels (RTLs) of serine-protease genes between WT and mutant strains. **(A)** Comparison of extracellular proteolytic activity of WT and Δ*Aoime2* mutants on casein plates. **(B)** Hyphal biomass of WT and Δ*Aoime2* mutants cultured in PL-4 medium for 7 days. **(C)** Proteolytic activity of WT and Δ*Aoime2* mutants quantified from 7-day-old PL-4 cultures. PMSF: serine-protease inhibitor, used at 5 mM. **(D)** RTLs of protease-encoding genes between WT and Δ*Aoime2* mutants at different time points. *CK* (RTL = 1) was used as the standard for statistical analysis of the RTL of each gene in the deletion mutants to that in the WT strain under a given condition. Error bars: SD from three replicates, asterisk: significant difference between mutant and WT (Tukey’s HSD, *p* < 0.05).

Serine proteases are closely related to the virulence in the NT fungi and entomopathogenic fungi (Yang et al., 2013). *A. oligospora* can produce serine proteases to immobilize the nematodes and degrade the nematode cuticle (Tunlid et al., 1994). To determine whether AoIme2 functions in serine-protease production, we qualitatively analyzed the proteolytic activities of the WT and Δ*Aoime2* mutant strains by using medium containing skimmed milk (Zhao et al., 2004), which revealed that proteolytic activity of the Δ*Aoime2* mutants was lower than that of the WT strain (Figure 6A). Quantification of hyphal biomass from 7-day-old PL-4 cultures of the Δ*Aoime2* mutants further revealed 9.9–10.3% decrease relative to that from WT cultures (Figure 6B), and total activity of extracellular proteases in the mutant cultures was found to be decreased by 32.2–38.6% as compared with that in WT cultures (Figure 6C). Notably, the proteolytic activities of the WT and mutant strains were inhibited by 90% when we added the serine-protease inhibitor PMSF (phenylmethylsulfonyl fluoride, 5 mM) (Figure 6C). Lastly, the transcription of almost all tested serine-protease genes was lower in the mutants than in the WT strain, and the transcripts of *PII* (*76g4*) and PII-like protease genes (*75g8* and *215g702*) in particular were significantly downregulated in the Δ*Aoime2* mutants (Figure 6D).

## Discussion

Inducer of meiosis 2, a non-classical MAPK-pathway molecule, has recently been identified in increasing numbers of fungal species and has been found to be involved in the regulation of multiple cellular processes, including vegetative growth, ascospore formation, environmental adaptation, and mating (Irniger, 2011). Here, we characterized AoIme2, an ortholog of *S. cerevisiae* Ime2, in the NT fungus *A. oligospora*. Similar to other Ime2 orthologs from yeast and filamentous fungi, AoIme2 and its orthologs share the conserved domains of MAPKs, but differ from the three classic MAPKs, Hog1-MAPK, Fus3/Kss1-MAPK, and Slt2-MAPK, in that they share differences in the phosphorylation site “-TXY-” and thus belong to the distinct Ime2-MAPK class (Garrido and Pérez-Martín, 2003; Schindler et al., 2003).

Disruption of the gene *Aoime2* caused defective mycelial growth in two Δ*Aoime2* mutant strains, and the aerial hyphae of the mutants were sparse. Our result agrees with what has been observed with the Δ*ime2* mutants of several filamentous fungi. In *Nomuraea rileyi*, deletion of *Nrime2* severely affected the growth rate of the strain and hyphal growth was delayed (Li, 2018). Similarly, inactivation of *crk1* (*ime2*) suppressed filamentous growth in *U. maydis* (Garrido and Pérez-Martín, 2003). Moreover, hyphal septa were increased here in Δ*Aoime2* mutants, as in the Δ*Nrime2* mutant in *N. rileyi* (Li, 2018), whereas mycelial and conidial cell nucleus numbers were markedly lower in the Δ*Aoime2* mutants than in the WT strain. Our results and the results of previous studies together suggest that Ime2 plays a critical role in hyphal growth, septum formation, and cell nucleus development in fungi.

We also recorded here a notable decrease (relative to WT) in the number of conidiophores and conidia in the Δ*Aoime2* mutants. Our result is similar to the result obtained after the deletion of *Scime2* in *S. cerevisiae*, which suggested that the Ime2 plays a unique role in the activation of sporulation-specific genes (Mitchell et al., 1990). However, a different result was reported in the case of the Δ*Nrime2* mutant in *N. rileyi*: deletion of *Nrime2* delayed the sporulation time but did not affect normal sporulation (Li, 2018). Furthermore, conidial morphology showed marked changes in the Δ*Aoime2* mutants here, and this is similar to two reported results: in *Schizosaccharomyces pombe*, disruption of Ime2-like protein kinase gene led to frequent production of asci containing an abnormal number of spores and spores of aberrant sizes (Abe and Shimoda, 2000); and in a budding yeast, where Ime2 is required for normal spore formation and spore-number control, abnormalities in spore formation were observed in diploid strains of the truncation mutant *ime2*Δ*C241* (Sari et al., 2008). Corresponding to the reduction in the number of conidia, the expression of several sporulation-related genes, particularly *abaA*, *fluG*, *velB*, and *aspB*, was substantially downregulated in the Δ*Aoime2* mutants during the conidiation stage. These genes are necessary for conidiation in *A. nidulans* and other filamentous fungi (Krijgsheld et al., 2013). For example, *fluG* is required for the synthesis of an extracellular sporulation-inducing factor and serves as a key activator of conidiation in *A. nidulans* (Seo et al., 2006); *aspB* is expressed during vegetative growth and asexual sporulation in *A. nidulans*, deletion of *aspB* is not lethal but results in delayed septation and greatly reduced conidiation (Momany and Hamer, 1997; Hernández-Rodríguez et al., 2012). Recently, we characterized the ortholog of VelB in *A. oligospora*, and we found that the Δ*AovelB* mutant displayed severe sporulation defects (Zhang et al., 2019). These results indicate that Ime2 plays a vital role in conidiation and asexual development in *A. oligospora* and other fungi.

The growth of Δ*Aoime2* mutants was inhibited under hyperosmotic stresses but was unaffected by oxidative and cell-wall-perturbing stresses. This is similar to the decreased growth of Δ*Nrime2* mutant after NaCl addition in the case of *N. rileyi* (Li, 2018). Furthermore, the protein encoded by *crk1* also plays a crucial role in environmental adaptation in *U. maydis*: when *crk1* was disrupted in *U. maydis*, cells were unable to appropriately respond to environmental stimuli (Garrido and Pérez-Martín, 2003). Moreover, we noted here that the hyphae of Δ*Aoime2* mutants were smoother than those of the WT strain, that partial hyphae were deformed, and that the cell wall of the trap cells of the Δ*Aoime2* mutants became loose; this suggested that AoIme2 regulates CWI and causes the increasing sensitivity to osmotic pressure. These results show that Ime2 is critical for osmotic-pressure adaptation in *A. oligospora* and other fungi.

Nematode-trapping fungi break down the nematode epidermis by producing serine proteases and related hydrolytic enzymes; this enables infection and helps in the assimilation of essential nutrients from the nematodes (Yang et al., 2013). In 1994, the serine protease PII was isolated from *A. oligospora*; the protease can immobilize *Panagrellus redivivus* and hydrolyze nematode-epidermis proteins (Tunlid et al., 1994). Subsequently, 24 genes encoding putative serine proteases were identified in the *A. oligospora* genome, and categorized into four subtilisin families (Yang et al., 2011). We found here that extracellular proteolytic activity of the Δ*Aoime2* mutants was markedly diminished and further that the proteolytic activity was potently inhibited by PMSF; this suggests that the main extracellular proteases are serine proteases. Furthermore, the reduction in protease activity coincided well with the transcriptional repression of almost all tested serine-protease genes in the mutant strains relative to WT. These results indicate that AoIme2 regulates the expression of serine-protease genes, and thus plays a crucial role in serine-protease production in *A. oligospora*.

Traps are essential for NT fungi to capture and infect nematodes. *Aoime2* disruption caused severe defects in trap formation: markedly fewer traps were present in Δ*Aoime2* mutants than in the WT strain, and most of the hyphal traps in the mutants did not form an intact ring; consequently, nematode capture by the Δ*Aoime2* mutants was substantially diminished relative to that by the WT strain. Similarly, *Nrime2* deletion in *N. rileyi* was reported to severely affect the virality caused by infestation (Li, 2018), and *crk1* in *U. maydis* was found to be required for pathogenicity, with the Δ*crk1* mutant being unable to cause infected plants to develop tumors (Garrido et al., 2004). Moreover, examination of the ED bodies in the traps produced by WT and Δ*Aoime2* mutant strains revealed that considerably fewer trap-cell ED bodies were present in the Δ*Aoime2* mutants. These results indicate that Ime2 plays a key role in the development of infectious structures required for fungal pathogenicity.

Mitogen-activated protein kinase signaling modules have been identified in diverse fungi and have been demonstrated to be involved in the regulation of multiple biological processes, such as conidiation, cell-wall biogenesis, and stress response (Xu, 2000; Zhao et al., 2007; Rispail et al., 2009). Previously, we characterized Slt2-MAPK in two NT fungi, *A. oligospora* and *Monacrosporium haptotylum*, and reported that *Slt2* disruption reduced mycelial growth, increased sensitivity to environmental stresses, and abolished the production of conidia and traps (Zhen et al., 2018). Here, we have identified an Ime2-MAPK ortholog for the first time from *A. oligospora* and have shown that AoIme2 is a multifunctional regulator, which may regulate the expression of phenotype-related genes, such as mycelial growth, conidiation, and trap formation genes; meanwhile, it also regulates the expression of serine-protease genes. Thus Aolme2 contributes to development and pathogenicity of *A. oligospora*.

## Conclusion

We characterized the Ime2-MAPK ortholog AoIme2 from the NT fungus *A. oligospora*. In this fungus, AoIme2 plays a critical role in mycelial growth, conidiation, and trap formation, as well as in septum formation and cell nucleus development. Moreover, AoIme2 functions in hyperosmotic-stress response and serine-protease production. Our results provide a basis for understanding the roles of Ime2-MAPK in other NT fungi, as well as for uncovering the mechanisms underlying mycelial development, trap formation, and lifestyle switching in NT fungi.

## Data Availability Statement

All datasets generated for this study are included in the article/Supplementary Material.

## Author Contributions

JKY and K-QZ conceived and designed the study. MX and JKY wrote the manuscript. MX, NB, KJ, and JLY conducted the experiments. DZ, YZ, and DL analyzed the data. JKY and XN revised the manuscript. All authors read and approved the final manuscript.

## Conflict of Interest

The authors declare that the research was conducted in the absence of any commercial or financial relationships that could be construed as a potential conflict of interest.

## Footnotes

1 https://blast.ncbi.nlm.nih.gov/Blast.cgi

2 http://www.expasy.ch/tools/pi_tool.html
