# Supplementary material for: Protein Kinase Ime2 Is Required for Mycelial Growth, Conidiation, Osmoregulation, and Pathogenicity in Nematode-Trapping Fungus Arthrobotrys oligospora
Source: Front Microbiol. 2020 Jan 14;10:3065. doi: 10.3389/fmicb.2019.03065 (PMC6971104; doi:10.3389/fmicb.2019.03065)
Supplement: Supplementary file 1 [file Data_Sheet_1.docx]

**Supplementary materials**

**1. Supplementary Figures**

**Supplementary Figure S1.** A neighbour-joining phylogenetic tree of the sequences orthologous to classic MAPKs family (Kss1/Fus3, Slt2, and Hog1) and Ime2 from diverse fungi. GenBank accession numbers are provided in brackets.


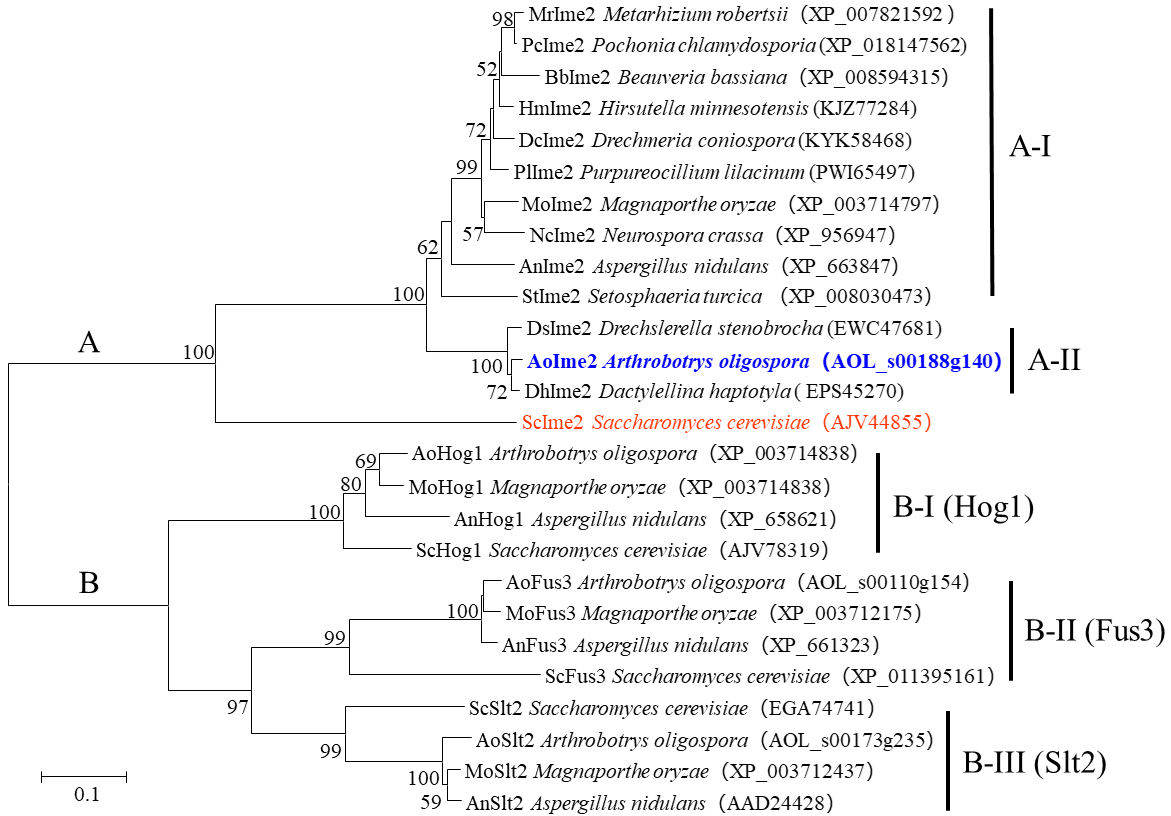


**Supplementary Figure S2.** Multiple alignment of the MAPK protein kinase domain (IPR000719) from different fungi. Areas shaded in black are conserved regions (100% similarity), Areas shaded in red have high degrees of homology (more than 75% similarity). The active site - D [L/I/V] K - and phosphorylation site - TXY -in different fungi were marked using underlines.


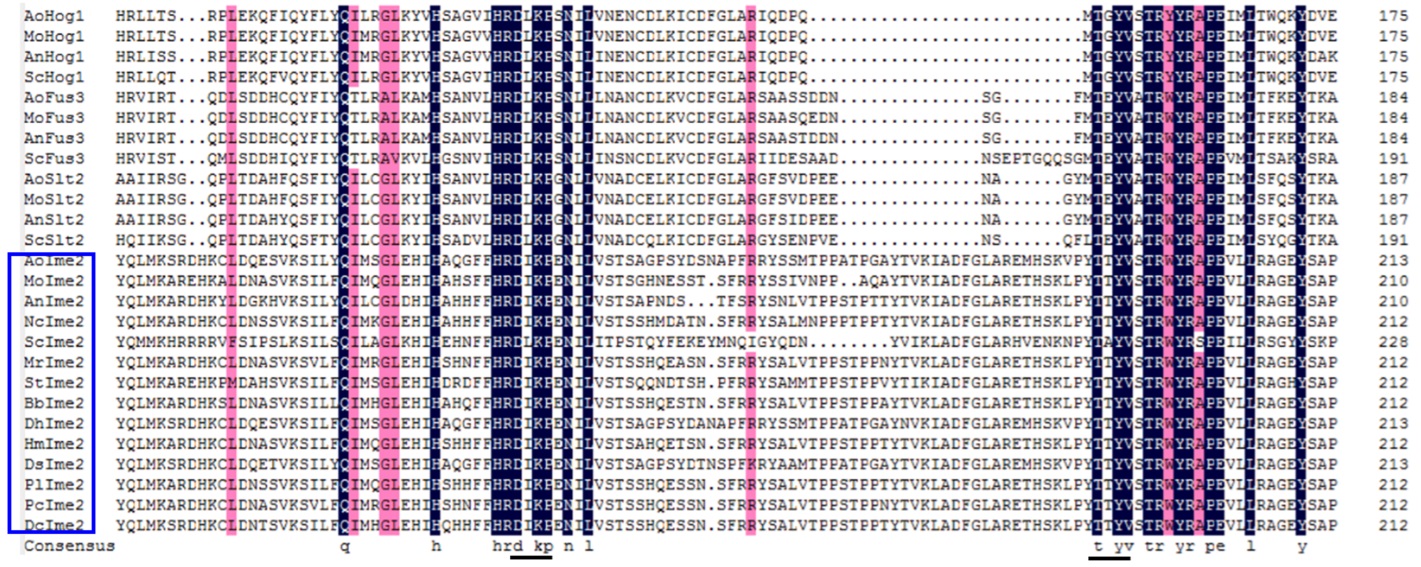


**Supplementary Figure S3.** Knock-out and verification of gene *Aoime2* in *A. oligospora*. **(A)** Diagrammatic sketch of homologous recombination of *Aoime2*. The homologous flanking sequences of the target gene, Southern blot probe, and the *Kpn*I restriction enzyme sites were marked. **(B)** Positive transformants were verified by PCR amplification. **C.** The Δ*Aoime2* mutants were confirmed by blotting analysis.

**
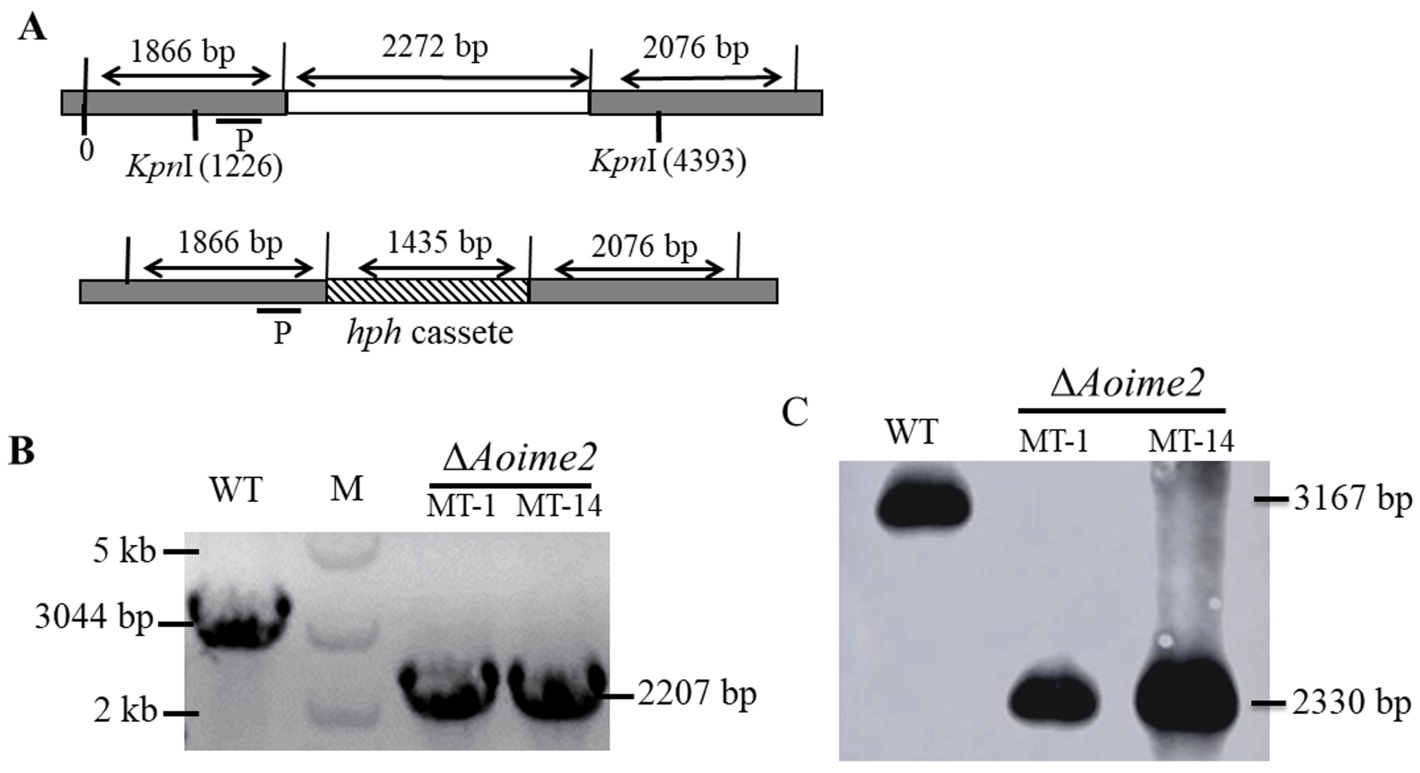
**

**Supplementary Figure S4.** Comparison of conidiophores between WT and Δ*Aoime2* mutant strains. Conidiophores and conidia of WT and mutants examined under a microscope.

**
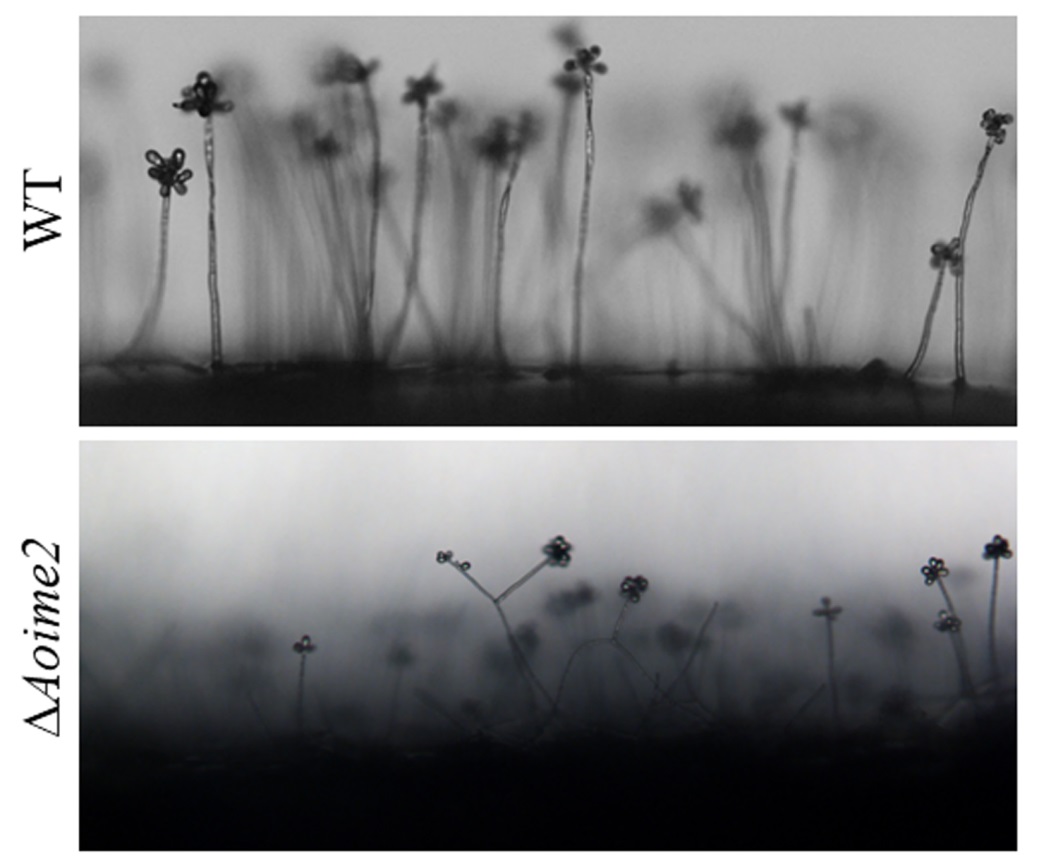
**

**Supplementary Figure S5.** Tolerance to oxidants and cell-wall-perturbing agents of WT and Δ*Aoime2* mutant strains. **(A)** Colony morphology under treatment with oxidants and cell-wall-perturbing agents: WT versus mutants. **(B)** RGI values of WT and mutants on TG plates supplemented with oxidants or cell-wall-perturbing agents.

**
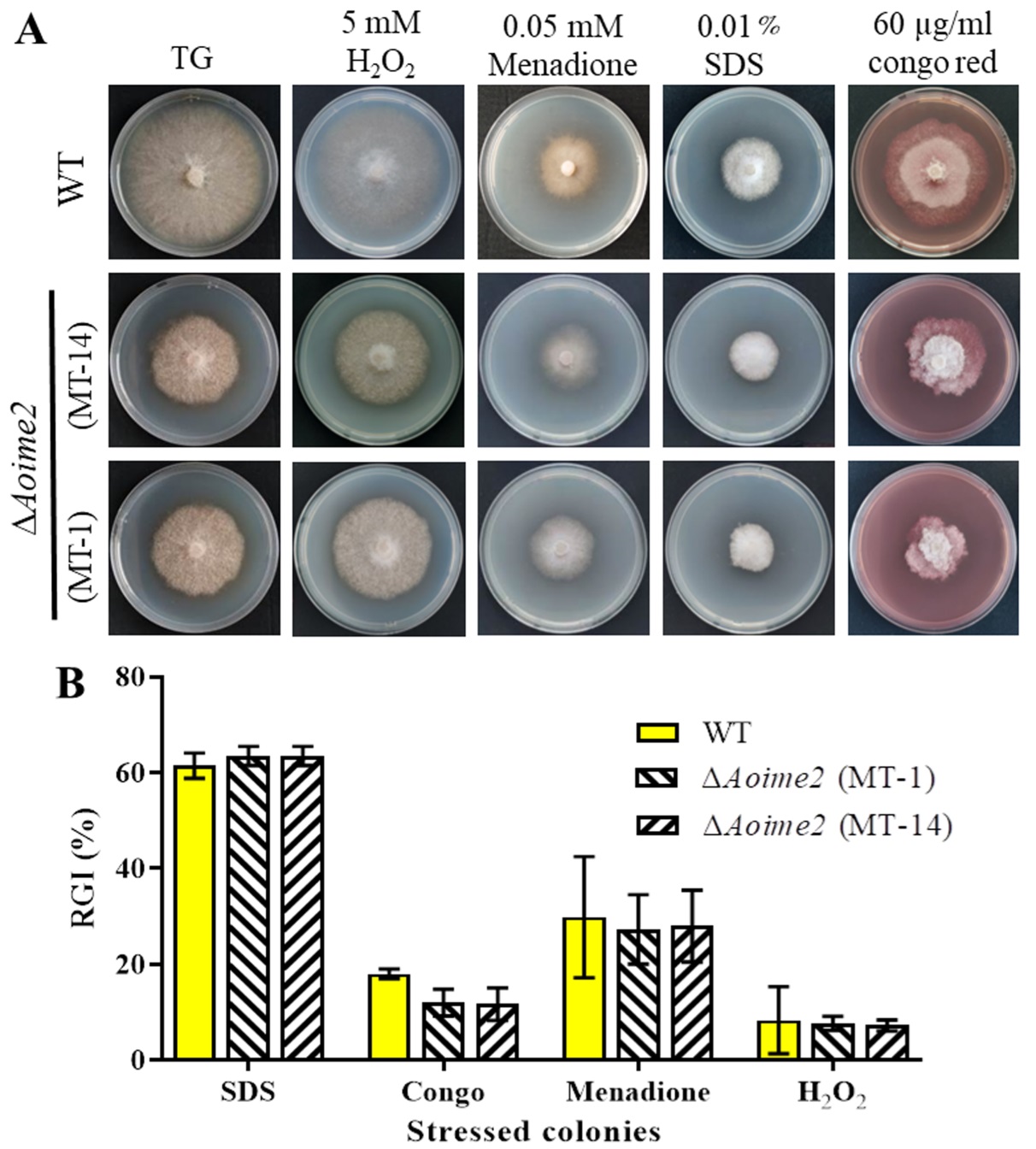
**

**2. Supplementary tables**

**Supplementary Table S1. List of primers used in this study.**

| Primers | Sequence (5′-3′) | Description |
| --- | --- | --- |
| AoIme2-5f | GTAACGCCAGGGTTTTCCCAGTCACGACGTGATGGGGTTGAAGTGAG | Amplify the*Aoime2* gene 5’ flank |
| AoIme2-5r | ATCCACTTAACGTTACTGAAATCTCCAACTCTCCGAACAGACGCACCA |  |
| AoIme2-3f | CTCCTTCAATATCATCTTCTGTCTCCGACTTTGGCGAGCACAACACTAC | Amplify the*Aoime2* gene 3’ flank |
| AoIme2-3r | GCGGATAACAATTTCACACAGGAAACAGCGAACCCCTGGCGAATCTGT |  |
| hphF | GTCGGAGACAGAAGATGATATTGAAGGAGC | Amplify the *hph* cassette |
| hphR | GTTGGAGATTTCAGTAACGTTAAGTGGAT |  |
| YZ140-5f | TTCGTGTCTGTTGGGTGATG | Verify the transformants |
| YZ140-3r | GAAAGGCTAAATCGGGGTC |  |
| probe5f | CTGGGGAAGAGTGAGTGAAAG | Make Southern blotting probe |
| probe3r | TGAAGATGACAGCCCGATC |  |

**Supplementary Table S2. Paired primers used for RT-PCR analysis, including sporulation-related genes and serine protease genes.**

| **Sporulation genes** | Sequence (5′-3′) | **Serine protease genes** | Sequence (5′-3′) |
| --- | --- | --- | --- |
| AOL_s00169g18  (*veA*) | 18-5F-AAGCTACACCCAATCAACGC | AOL_s00112g42 | 42-5F-CTGGCTCTTGGCCTACTTTG |
|  | 18-3R-TTGCGATGCTGACGATCTTG |  | 42-3R-AGGAGGTTGACGGTCTCCTT |
| AOL_s00007g157 (*flbC*) | 157-5F-CTCTCCGGCAAAGACAATCG | AOL_s00075g8 | 8-5F-TTGCTACTTTACTGCCCCTTG |
|  | 157-3R-GTCGACTGAGGATAGTAGCT |  | 8-3R-TCTTCAGCTTGAGTCCGGTT |
| AOL_s00075g211 (*nsdD*) | 211-5F-ATTACGGCCGCCTAGTAGTC | AOL_s00215g702 | 702-5F-GTCGCCGCTGACTTAACTGT |
|  | 211-3R-CTCGTTTGGACCTGGTTGTG |  | 702-3R-ATAATTGCTGATTCGCTGGG |
| AOL_s00043g361 (*fluG*) | 361-5F-GATTCCAGTCCCGTGAATTC | AOL_s00176g95 | 95-5F-CCCAAAAAGAATGCACCACT |
|  | 361-3R-GCTAAGGAGAGGATGGGCAT |  | 95-3R-AGAGGCGGGAAAATACCTGT |
| AOL_s00006g570 (*rodA*) | 570-5F-GCGGATCCAACATGAAGCTT | AOL_s00076g4 (*PII*) | 4-5F-CCATGGTGTTGGAAGGAAAT |
|  | 570-3R-GGTTGACAACTGGGATGCTG |  | 4-3R-GTCTGTTCAGCGTACGTAGT |
| AOL_s00080g63 (*abaA*) | 63-5F-AACTTTATGCGCCTTGTCGT | AOL_s00078g136 | 136-5F-ACACTTGCCCATTTCACTCC |
|  | 63-3R-TTGGCTAGGTGGTCTGTACG |  | 136-3R-GCTGGGTTTCACAACATCCT |
| AOL_s00054g811 (*velB*) | 811-5F-ATTCCGCAACTTCTCCCTCA | AOL_s00188g273 | 273-5F-GTGGATGAAACCCGGATATG |
|  | 811-3R-GGCATGTTTGGATTCTGGGG |  | 273-3R-CTCCCACTATCACCGCAACT |
| AOL_s00215g893 (*aspB*) | 893-5F-ATACCGCCAACACCCTCTAC |  |  |
|  | 893-3R-AACCATCTTCATCTCGGCCT |  |  |
| **β-tubulin gene** | Sequence (5′-3′) |  |  |
| AOL_s00076g640  (*tub*) | tubA-F-CCACCTTCGTCGGTAACTC |  |  |
|  | tubA-R-TCGTCCATACCCTCACCAG |  |  |
